# Prevalence of Perforated Graft in Underlay and Pop-in Technique Myringoplasty

**DOI:** 10.31729/jnma.4709

**Published:** 2019-12-31

**Authors:** Bhuwan Raj Pandey, Poonam KC

**Affiliations:** 1 Department of ENT-HNS, Lumbini Medical College, Palpa, Nepal; 2 Department of ENT-HNS, Ganeshman Singh Memorial Hospital and Research Center, Lalitpur, Nepal

**Keywords:** *myringoplasty; pop-in*, *trans-tympanic*, *underlay*

## Abstract

**Introduction:**

Underlay technique myringoplasty is most commonly used technique to repair tympanic membrane perforation by temporalis fascia graft and Trans-tympanic pop-in technique is an another technique that allows temporalis fascia graft placement medial to tympanic membrane remnant through the perforation without the need for tympano-meatal flap elevation. This study was undertaken to find the prevalence of graft failure in underlay and Pop-in Technique Myringoplasty.

**Methods:**

This descriptive cross-sectional study was done at Manipal teaching hospital, Pokhara, Nepal and comprises of 86 patients between January 2014 and June 2015. Patients undergoing Underlay Trans-canal approach by tympano-meatal flap elevation and Trans-canal, Trans-tympanic pop-in technique were included. Sample size calculation was done and convenient sampling method was applied. Point estimate at 95% CI was done for binary data along with frequency and proportion. The descriptive statistical analysis was done.

**Results:**

The prevalence of perforated graft was 15 (17.4%) at 95% Confidence Interval (39.75-60.25%). In underlay technique there were 8 (18.60 %) perforation and 35 (81.39%) were intact while in pop-in technique there were 7 (16.27%) perforation and 36 (83.72%) were intact. The postoperative mean Pure Tone Average (PTA) of underlay was 9.53 and pop-in was 8.31. The mean Pure Tone Average (PTA) gain after underlay technique was 16.095 and pop in technique was 16.87.

**Conclusions:**

Prevalence of perforated graft in Trans-tympanic pop-in myringoplasty and underlay myringoplasty gives similar hearing & graft uptake results when compared with similar studies.

## INTRODUCTION

Chronic Suppurative Otitis Media (CSOM) is defined by otorrhoea of at least six weeks duration in the presence of a chronic tympanic membrane perforation.^1^ Myringoplasty is a simple surgical repair of a tympanic membrane perforation without ossicular reconstruction. The purpose of the operation includes closure of the perforation and improvement in hearing levels.^2^

Around 80% tympanic membrane perforation heal spontaneously while the remaining needs to be repaired.^3^ Surgical outcome depends on location and size of perforation, chronicity, age, gender, techniques and experience of the operating surgeon.^4^

Long-standing tympanic membrane perforations may cause hearing loss and middle ear infection even if they are small.^5^ The trans-tympanic pop-in technique is an alternate technique that allows temporalis fascia graft placement medial to tympanic membrane remnant through the perforation without the need for tympano-meatal flap elevation. It is easier to learn, takes lesser time and cost-effective.

The aim of this study was to find the prevalence of perforated graft in underlay and Pop-in Technique Myringoplasty.

## METHODS

This was a descriptive cross-sectional study carried out in Manipal Teaching Hospital from January 2014 to June 2015. Ethical approval was taken from Institutional Review Committee.

Sample size calculation was done as follows:

$$\begin{array}{ll} \text{n} & ={\text{Z}}^{\text{2}}\times \text{(p}\times \text{q)}/{\text{e}}^{\text{2}}\\ & ={\text{(1.64)}}^{\text{2}}\times \text{0.5}\times \text{0.5}/{\text{(0.1)}}^{\text{2}}\\ & =64 \end{array}$$

where,

n = sample size
Z = confidence interval at 90% (1.645)
p = 50% prevalence
q = 1-p
e = margin of error, 10%
So, the final sample size is 64.

Total 86 patients were included by convenient sampling method with written informed consent and divided into two groups based on surgical technique.

Inclusion criteria are patient having Chronic Suppurative Otitis Media, tubotympanic type with small to medium size central perforation, normal external auditory canal, aged between 15 to 45 years, Pure Tone Average of the ear to be operated should be between 20 to 45 decibels, should be without discharge for at least 4 weeks before surgery, no sensorineural hearing loss (adequate cochlear reserve must be present), operated ear should be the worse hearing ear, any focus of infection in the nose, paranasal sinuses and throat was ruled out and Eustachian tube function should be normal. Patients with large and sub-total tympanic membrane perforation were excluded.

The collected data was entered in SPSS version 17 and the descriptive statistical analysis was done. Frequency and percentage were calculated for binary data and standard deviation and mean were calculated in continuous data.

## RESULTS

The prevalence of perforated graft was 15 (17.4%). Among the total number patient, minimum age in this study was 15 years old and while oldest patient age was 45 with mean age is 23.48±7.603. This study showed that maximum patient were in age group of 15-25yr and lowest in 36-45yr age group (Table 1).

**Table 1:** Age distribution of patients.

Age distribution in years	Underlay technique	Pop-in technique	n (%)
15-25	31	31	62 (72.09)
26-35	7	7	14 (16.27)
36-45	5	5	10 (11.62)

Among the patient having CSOM, male were 45 (52.3 %) and female were 41 (47.7 %) and this study showed that patient presenting in our OPD male were more than female.

The post-operative pure tone average mean of underlay technique was 9.53±6.06 and 8.31±4.48 for pop-in technique.

**Table 3:** Post-operative PureTone Average ofoperated ear.

Pure tone average (dB)	Underlay Myringoplasty n (%)	Pop-in myringoplasty n (%)
0-10	33 (76.74)	35 (81.39)
11-15	4 (9.30)	2 (4.65)
16-20	3 (6.97)	4 (9.30)
>20	3 (6.97)	2 (4.65)
Total	43 (100)	43 (100)

The PTA gain between underlay technique mean was 16.095±5.28 and pop-intechnique mean was 16.87±4.14.

**Table 4:** Post-operative air-bone gap closure (gain).

Air-Bone Gap Closure (dB)	Underlay Myringoplasty n (%)	Pop in myringoplasty n (%)
0-10	4 (9.30)	3 (6.97)
11-20	31 (72.09)	32 (74.41)
21-30	8 (18.60)	8 (18.60)
Total	43 (100)	43 (100)

In underlay technique there were 8 (18.60%) perforation and 35 (81.39%) were intact and in pop-in technique there were 7 (16.27%) perforation and 36 (83.72%) was intact.

**Figure 5:**
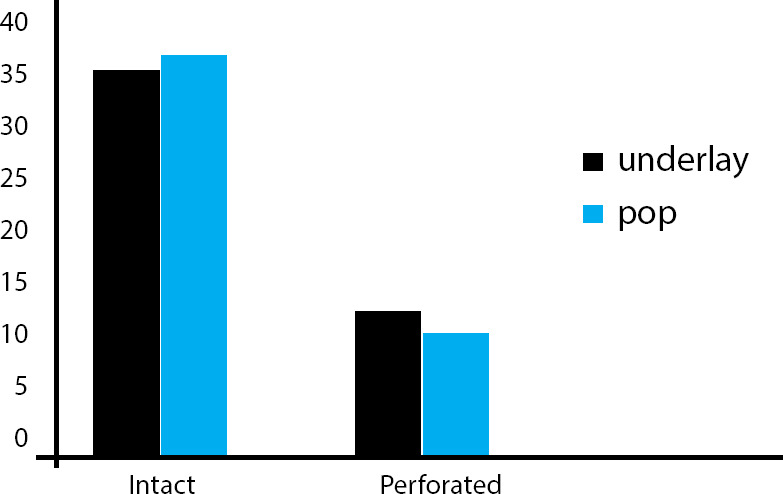
Post-operative graft status.

The PTA gain between underlay technique mean was 16.095±5.28 and popin technique mean was 16.87±4.14.

**Figure 6:**
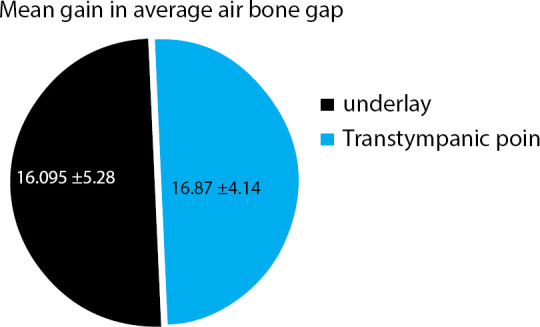
Mean gain in average air-bone gap after surgery.

## DISCUSSION

Total 113 patient with COM Mucosal type were presented in our hospital from January 2014 to June 2015 out of which 10 patient had total perforation and 8 have subtotal perforation and 5 patient excluded from surgery due to medical unfitness and 4 patient refused for surgery. The remaining 86 patients underwent myringoplasty. These patients fulfilled the inclusion criteria mentioned above.

Out of 86 patients there were 43 cases for underlay myringoplasty and 43 cases for transtympanic popin myringoplasty by permeatal approach. This study compares the results of graft status and hearing outcome at 6 months between underlay myringoplasty with pop-in myringoplasty by transcanal permeatal approach.

In our study mean age is 23.48±7.603. This study showed that 72.09% patient were in age group of 15-25 years. The number of male patient in our study was more i.e. 52.3% (M:F=1.097:1) and higher incidence in a lower age group is due to this diseases is more among pediatrics age group.

In this study the tympanic membrane perforation closure at 6 months after surgery in the underlay method was 81.39% and pop-in method was 83.72%. This study showed that for small to medium size perforation the repair of tympanic membrane with temporalis fascia by underlay technique is almost similar to transtympanic pop-in technique. This does not show any significant difference between these two techniques in graft status at 6 months.

The post-operative mean pure tone average of underlay technique was 9.53±6.06 and 8.31±4.48 for popin technique. The mean PTA gain after underlay technique was 16.095±5.28 and popin technique was 16.87±4.14.

Alzoubi, et al.^6^ reported perforation closure rates of 78% transcanal tympano-meatal flap and 72% transtympanic myringoplasty respectively.

El-Guindy,^7^ reported a 91% success rate with transtympanic myringoplasty in adults using a rigid endoscope and perichondrium as the graft material.

Naganuma, et al.^8^ reported a 78% success rate in adults using homologous temporalis fascia as the graft material by the transtympanic method, the procedures being done under local anesthesia.

Srinivasan, et al.^9^ described trans-tympanic ‘push through’ technique using temporalis fascia in 40 children with the rate for perforation closure as 77.5% at 6 months. They reported no significant relationship between the site of perforation and successful outcome.

Singh GB, et al.^10^ reported 25 cases of trans-tympanic myringoplasty with a success rate of 84% in terms of perforation closure. They also opined that surface area of perforation did not influence the result, however anterior perforations showed poor graft uptake rates.

The limitations of the study is that less sample size was taken as well as longer follow-up time was needed.

## CONCLUSIONS

Prevalence of perforated graft in Trans-tympanic pop-in myringoplastyand underlay myringoplasty gives similar hearing & graft uptake results when compared with similar studies.

In order to increase the quality of the study, more sample size is recommended to be taken.
